# Cell type specificity of tissue plasminogen activator in the mouse barrel cortex

**DOI:** 10.1016/j.dib.2015.06.008

**Published:** 2015-06-25

**Authors:** Philip Chu, Eric Chen, Adesh Bajnath, Joshua C. Brumberg

**Affiliations:** a Neuropsychology doctoral program, The Graduate Center, City University of New York (CUNY), United States; b University of California, Santa Cruz, United States; c Neuroscience Biology Ph.D. subprogram, The Graduate Center, CUNY, United States; d Department of Psychology, Queens College, CUNY, United States

## Abstract

We provide data in this article related to (C.C. Chen et al.,. Neurosci. Lett., 599 (2015) 152–157.) [1] where the expression of tissue plasminogen activator (tPA) is expressed by the whisker representation in the somatosensory cortex. Here, we provide immunocytochemistry data indicating that tPA is expressed by putative excitatory neurons as well as parvalbumin+ interneurons but not by somatostatin+ inhibitory interneurons. We also provide data showing that microglia do not normally express high levels of tPA, but upregulate their levels following cortical penetration with a recording electrode.

Specifications table

**Table:** 

Subject area	Neuroscience
More specific subject area	Barrel cortex, tissue plasminogen activator cell type specificity
Type of data	Image (immunohistochemistry)
How data was acquired	Compound fluorescent microscope (Olympus Bx-51) and confocal microscope (Olympus Fv10i).
Data format	Raw
Experimental factors	Brain tissue was obtained from control, untreated brains (Fig. 1) or following in vivo recordings under xylazine/ketamine anesthesia (Fig. 2).
Experimental features	Coronal sections from the barrel cortex were labeled using primary antibodies raised against neurogranin, parvalbumin, somatostatin, Iba1 (microglia), and tissue plasminogen activator. Non-specific blocking, tissue permeablization and signal amplification was applied using the appropriate secondary antibodies. 1+hours of cortical electrode penetration was conducted using a tungsten microelectrode in an adult anesthetized CD-1 mouse.
Data source location	Flushing, NY, United States of America
Data accessibility	Data within this article

Value of the data

• The immunohistochemistry data shown here can be compared to data in other model systems to better understand tPA expression at the global and cellular level.
• tPA has been shown to activate the brain׳s immune system [4], double-labeling of microglia (brain׳s immune system cells) and tPA evidence this.
• Knowing tPA specific activation patterns is important in understanding how the nervous system regulates cell death [6,3], and cortical plasticity [2,5].

## 1 Data, experimental design, materials and methods

**Fig. 1:**
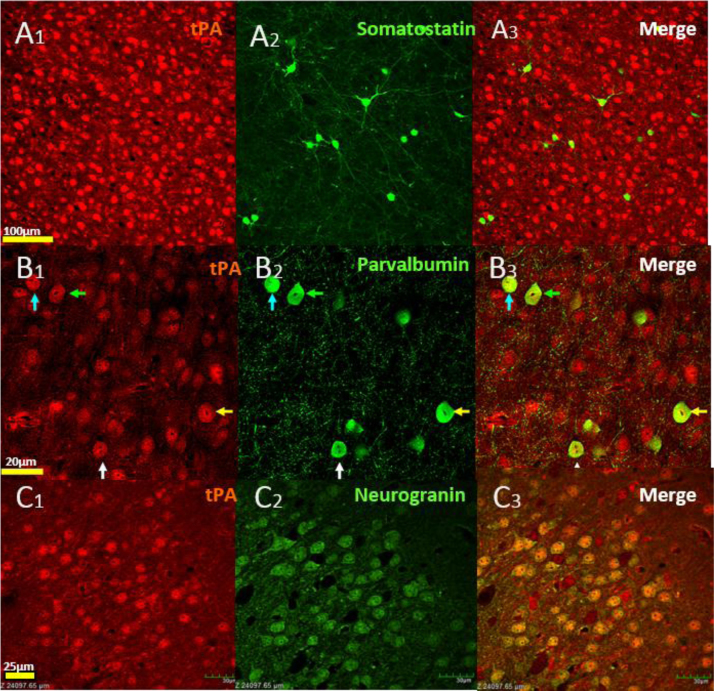
Neuron specific expression of tPA in the somatosensory barrel cortex. Immunofluorescence for tPA (A1,B1,C1) does not colocalize with somatostatin+interneurons (A2,A3), but is strongly expressed by Parvalbumin+interneurons (B2,B3; Green and blue arrows) and putative excitatory neurons detected with Neurogranin (C2,C3).

**Fig. 2:**
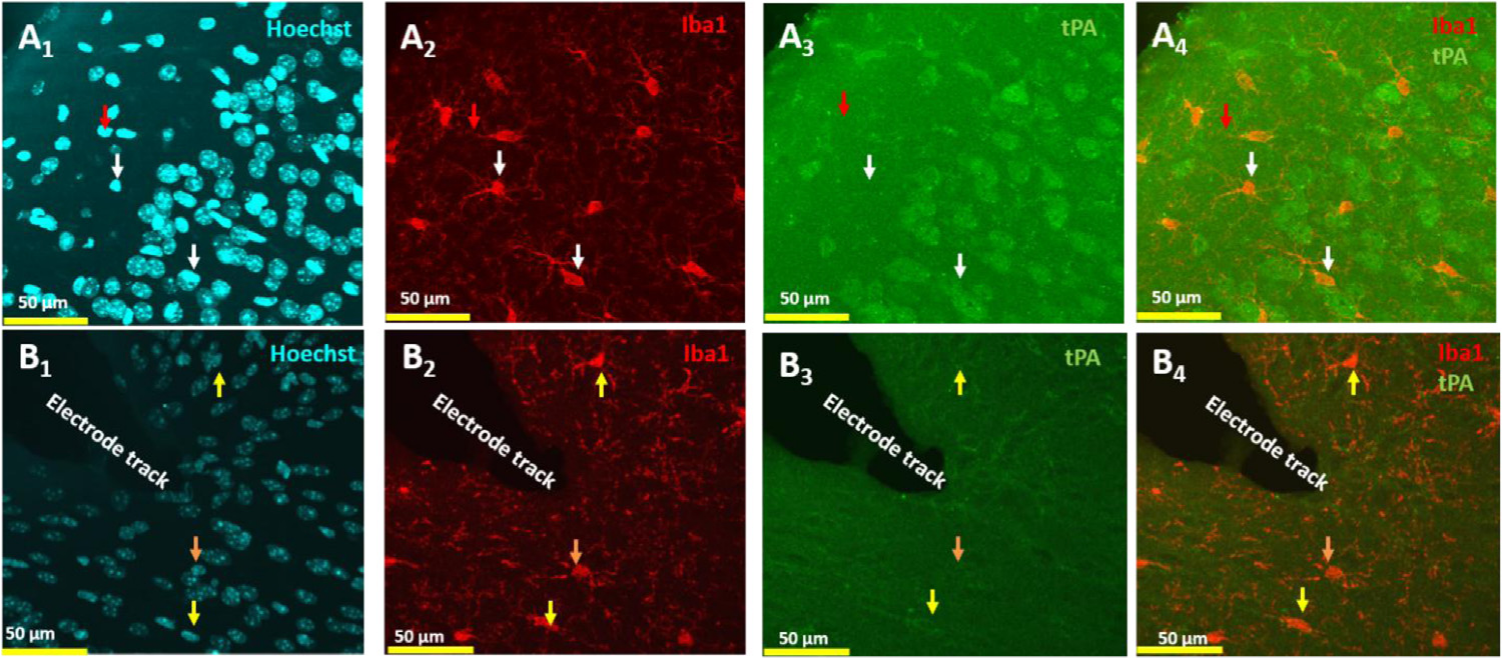
Expression of tPA in microglia is context dependent. tPA typically is not expressed by microglia labeled with Iba1 (White arrows; A1–A4), nor other small nuclear cells in the barrel cortex (red arrow; A1–A4). However, following lesion induced by 1+hours of cortical electrode penetration (upper left in all images is pia), neuronal tPA appears to move from intracellular expression to membrane expression (compare A3–B3). Microglia also appears to take on ameboid morphologies (B2, B4) and occasionally express tPA on their membranes (yellow arrows; B3, B4). To confirm this we computed Pearson correlation coefficients to quantify the number of double labeled pixels in control and recorded from hemispheres. In control hemispheres Pearson׳s coefficient was 0.094 and following recordings the coefficient increased significantly to 0.45 (*p*<0.03). Our results show that tPA is expressed in a cell type and situation specific manner.

We have shown that tPA expression in the somatosensory cortex is dependent on the sensory experience of the animal [1]. Here, using double immuno labeling for tPA and makers of specific cell populations we provide the data showing cell type-specific tPA expression (Fig. 1) and that double-labeling patterns are not fixed (Fig. 2).

### 1.1 Cortical penetration

Adult male CD-1 mice (>P30) was anesthetized using a 130/10 mg/kg ketamine–xylazine cocktail. After the animal reached sufficient anesthetic state (non- responsive to a noxious stimuli; toe pinch), it was head-fixed in a stereotaxic apparatus (Kopf instruments) followed by a small craniotomy over the barrel cortex (−2.00 A.P, +3.50 M.L relative to Bregma). Coordinates were obtained from a mouse brain atlas (George Paxinos 2012). A precision micro-manipulator (Kopf Instruments) was then used to insert a high-impedance tungsten microelectrode (3.7 MΩ; FHC) into the cortex such that neural activity from thalamic recipient Layer IV neurons was recorded to disc using a multi-channel data acquisition device (Molecular devices) at a rate of 30 kHz for 1.5 h. Multiple cortical penetrations were made at a depth of 450 μm in order to evaluate whisker evoked responses in layer 4 of the barrel cortex.

### 1.2 Immunocytochemistry

Animals were deeply sedated using intraperitoneal injection of Euthasol (0.1 ml, Virbac AH, Inc.) until unresponsive to toe pinch. Transcardial perfusion was then conducted with 0.01 M phosphate buffer saline (0.01 M PBS) followed by 4% paraformaldehyde (PFA) in 0.01 M PB. Fixed brains were kept overnight in PFA at 4 °C, then sectioned using a vibratome (Leica) at 60 μm at room temperature. Free floating sections were rinsed 3× for 10 min each in 0.01 M PBS before and after each of the following steps. All double-immunofluorescence experiments were performed with sequential staining instead of mixing primary antibodies in cocktail. For double-labeling with microglia, tissue was permeabilized and blocked using 0.5% Triton-X and 5% normal donkey serum (NDS) for 1 h at room temperature followed by 1:1000 Iba1 primary antibody (ABCAM, host goat) in 0.01 M PBS and 2% NDS for three nights overnight at 4 °C. Slices were then incubated in a cocktail of 2% NDS and 1:200 Alexa 594 Antigoat (Jackson Immuno) secondary antibody for 2 h. Following incubation in 0.5% Triton-X and blocking in 5% normal serum for 30 min, slices were incubated in 1:100 tPA primary antibody(American Diagnostica; host rabbit) with 2% NDS for three nights overnight. Slices were then incubated in a cocktail of Dylight 488 Antirabbit (Jackson Immuno) and 2% NDS for 2 h at 37 °C. Lastly, the brain tissues were counterstained with Hoechst (Sigma-Aldrich, dilution 1:10,000, final solution 0.12 µg/ml) for 30 min, mounted onto gelatin subbed slides and cover slipped using Vectashield (Vector Laboratories). Negative controls were conducted by following the aforementioned procedures, but leaving out either the primary antibodies, or the secondary antibodies. No non-specific background labeling of cells were detected in the control tissue.

For neuronal double-labeling experiments, somatostatin were identified with intrinsic GFP from GIN mouse (strain FVB-Tg(GadGFP)45704Swn/J, purchased from Jackson Laboratory, Bar Harbour, Maine). For the double-immunohistochemical portion of the study, a separate group of animals (*n*=3, P30) were used to investigate the colocalization profile of tPA with parvalbumin and neurogranin immunopositive cells. The parvalbumin and neurogranin immunostaining was conducted as follows: Tissues were blocked in 5% normal donkey serum with 0.3% Triton X in 0.01 M PBS, then incubated in anti-parvalumin (host mouse, Sigma-Aldrich, dilution 1:2000) or anti-neurogranin (host rabbit, EMD Millipore, dilution 1:500) for 24 h at 4 °C in 0.01 M PBS. After rinsing in 0.01 M PBS, the tissues were submerged in a secondary antibody (Parvalbumin staining: Alexa 488-conjugated donkey anti-mouse, excitation: 519 nm, dilution 1:200; Jackson Immuno Research. Neurogranin staining: Alexa 488-conjugated donkey anti-rabbit, excitation: 519 nm, dilution 1:200) for 2.5 h in room temperature. Afterwards, tPA immunofluorescent staining protocol was followed as previously described, with the exception of replacing the primary antibody (Molecular Innovations, host goat, 1:200 dilution) and fluorophore to streptavidin with Alexa 647 (excitation: 650 nm; Invitrogen). Last, Hoechst staining was performed, and brain slices were extensively washed (60 min minimum), air dried, dipped in distilled H20, and covered-slipped as previously described.

### 1.3 Confocal microscopy

Imaging was conducted using a confocal microscope (Olympus Fv10i) using the Alexa 594 (Ex:559 nm Em: 618 n), FITC (Ex: 473 Em:519 ) and DAPI filter (Ex:405 Em:461 ) using a 60× lens (NA: 1.35). 0.5 μM z-step stacks were taken and the data was analyzed offline. The colocalization function was used to determine the degree of overlap between the different channels at individual pixels. Colocalization analysis yielded a Pearsons correlation for the pixel intensities between the two channels (Vertical threshold: 1500, Horizontal threshold: 1500).
